# Short‐term removal of exercise impairs glycemic control in older adults: A randomized trial

**DOI:** 10.14814/phy2.15591

**Published:** 2023-01-25

**Authors:** Leryn J. Reynolds, Troy M. Williams, Joel E. Harden, Hannah M. Twiddy, Monica L. Kearney

**Affiliations:** ^1^ Old Dominion University Norfolk Virginia USA; ^2^ Eastern Virginia Medical School Norfolk Virginia USA; ^3^ Southeast Missouri State University Cape Girardeau Missouri USA

**Keywords:** aging, glucose, glycemia, inactivity

## Abstract

Postprandial glycemia (PPG) predicts cardiovascular disease, and short‐term physical inactivity increases PPG in young, active adults. Whether this occurs in older, active adults who may be more prone to bouts of inactivity is unknown. This study determined if postprandial interstitial glucose (PPIG) was impaired in active older adults following the removal of exercise for 3 days (NOEX) compared to active young adults. In this randomized, crossover study, 11 older (69.1 ± 1.9 years) and 9 young (32.8 ± 1.8 years) habitually active (≥90 min/week of exercise) adults completed 3‐days of NOEX and 3‐days of normal habitual exercise (EX), separated by ≥1 week. Diet was standardized across phases. Glycemic control (3‐day average) was assessed via continuous glucose monitoring during both phases. Significant main effects of age and phase were detected (*p* < 0.05), but no interaction was found for steps/day (*p* > 0.05) (old EX: 6283 ± 607, old NOEX: 2380 ± 382 and young EX: 8798 ± 623, young NOEX: 4075 ± 516 steps/day). Significant main effects of age (*p* = 0.002) and time (*p* < 0.001) existed for 1‐h PPIG, but no effect of phase or interactions was found (*p* > 0.05). Significant main effects (*p* < 0.05) of age (old: 114 ± 1 mg/dl, young: 106 ± 1 mg/dl), phase (NOEX: 112 ± 1 mg/dl, EX: 108 ± 1 mg/dl), and time (0 min: 100 ± 2, 30 min: 118 ± 2, 60 min: 116 ± 2, 90 min: 111 ± 2, 120 min: 108 ± 2 mg/dl) in 2‐h PPIG were detected, but no interaction was found (*p* > 0.05). However, only significant main effects of phase (NOEX: 14 ± 1 and EX:12 ± 1, *p* > 0.05) were found for 24‐h blood glucose standard deviation. Older adults appear to have impaired glycemic control compared to young adults and exercise removal impairs glycemic control in both populations. Yet, the impairment in glycemic control with exercise removal is not different between old and young adults.

## 1 INTRODUCTION

The global prevalence of type 2 diabetes continues to rise. By the year 2030, an estimated 7079 individuals per 100,000 will have type 2 diabetes (Khan et al., 2020). Diabetes is the ninth leading cause of death worldwide and contributes substantially to the economic burden of healthcare costs. Approximately 1 in 4 older adults has diabetes (Centers for Disease Control and Prevention (CDC), 2020) and this is linked to reduced functional status and increased risk of institutionalization. Further, older adults with diabetes are at an increased risk of cardiovascular complications and premature death (Brown et al., 2003). Thus, understanding mechanisms that lead to diabetes in older adults are imperative. Traditionally, glycosylated hemoglobin (HbA1c) or fasting glucose concentrations are measured as indicators of diabetes status (Guo et al., 2014). However, elevated postprandial blood glucose (PPG) is a common attribute of individuals with diabetes (Chang & Halter, 2003) and even precedes type 2 diabetes development (Bock et al., 2006). While acute changes in PPG may not lead to sustained chronic changes per se, understanding the time course when alterations in PPG occur may be beneficial in mitigating chronic glycemic excursions and preventing or slowing the development of type 2 diabetes.

Advancements in glucose monitoring make assessing free‐living postprandial blood glucose levels convenient and feasible (Grant & Golden, 2019). Continuous glucose monitoring systems assess blood glucose levels 24 h a day and have been demonstrated to enhance diabetes management (Beck et al., 2017). Both acutely and chronically, physical activity and/or exercise is a powerful modulator of glycemic control (Boule et al., 2001; Mikus, Oberlin, Libla, Boyle, & Thyfault, 2012). Not only does muscle contraction stimulate glucose uptake into skeletal muscle in an insulin‐independent manner during exercise (Jessen & Goodyear, 2005), but both acute and chronic exercise enhances insulin‐mediated glucose uptake (Wojtaszewski & Richter, 2006). The effects of a single bout of exercise to enhance insulin‐mediated glucose uptake into skeletal muscle appear to last for ~2 days (Burstein et al., 1985), which demonstrates the need for daily physical activity. A number of studies have examined how short‐term increases or decreases in physical activity/exercise modulates postprandial blood glucose levels acutely. Mikus et al. (Mikus, Oberlin, Libla, Boyle, & Thyfault, 2012) demonstrated that only 7 days of exercise in previously sedentary individuals with type 2 diabetes improved the postprandial blood glucose response. We (Reynolds et al., 2015) and others (Mikus, Oberlin, Libla, Taylor, et al., 2012) have demonstrated that short‐term reductions in physical activity (3 and 5 days) worsen glycemic control in young, college‐aged individuals. Further, McGlory et al. (2016) demonstrated that laboratory‐based indices of glycemic control, HOMA‐IR, and MATSUDA index, were impaired following 2‐weeks of physical inactivity in older adults. However, these laboratory‐based measurements do not assess glycemic variability under free‐living conditions. Thus, the day‐to‐day variability of blood glucose concentrations and the magnitude of postprandial hyperglycemia are largely understudied in the older population in response to acute physical inactivity or exercise reduction, particularly in free‐living conditions. The purpose of this study was exploratory in nature and aimed to examine if the alterations in glycemic control [PPIG, PPIG area under the curve (AUC), blood glucose standard deviation, and maximum glucose values] in older adults who exercise were different compared to young adults who exercise, in response to short‐term removal of exercise. We hypothesized that compared to young adults, older adults would have greater increases in PPIG, blood glucose standard deviation, and maximum glucose values, after the removal of exercise. To assess glycemic control under free‐living conditions, we utilized continuous glucose monitoring systems (CGMS) to determine PPIG, PPIG AUC, blood glucose standard deviation, and maximum glucose values. Participants consumed standardized meals during each of the study phases.

## 2 METHODS

### 2.1 Study design

This randomized, cross‐over, exploratory study design included 20 participants (9 young, active participants and 11 older, active participants). Participants had glycemic control assessed via CGMS (iPro2, Enlite Sensors, Medtronic Plc.) for 3 days while performing their normal habitual exercise (EX) and for 3 days while refraining from habitual exercise (NOEX). All participants completed 1 exercise session on each of the 3 days during the EX phase, except 1 young subject who only participated in exercise on days 1 and 3 of the study intervention.

**FIGURE 1:**
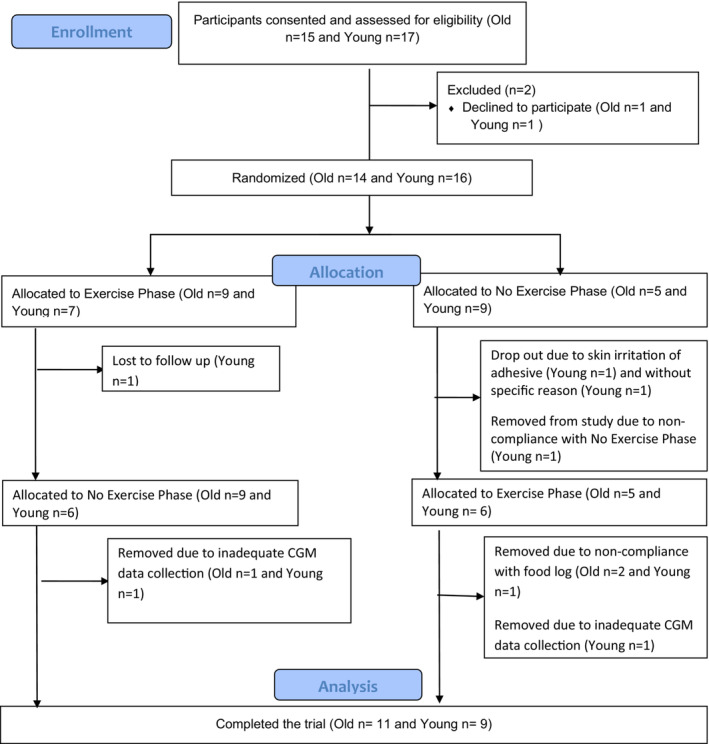
Flow diagram for study enrollment, participant allocation, and data analysis

The primary outcome of glycemic control was 1‐h PPIG. We also measured 2‐h PPIG, 2‐h PPIG AUC, 24‐h blood glucose maximum, and 24‐h blood glucose standard deviation. Subjects were randomized by phase order using an online randomization tool (Research Randomizer Version 4.0) (Urbaniak & Plous, 2013) to prevent potential bias of phase order. Each phase was separated by at least 1 week. We (Reynolds et al., 2015) and others (Mikus, Oberlin, Libla, Taylor, et al., 2012) have previously used short‐term (3 and 5 days) inactivity models to examine changes in free‐living glycemic control in young populations and found this to be a sufficient amount of time to alter postprandial blood glucose levels. Thus, we also implemented a 3‐day removal of exercise phase for this study. Participants wore an Actigraph accelerometer (wGT3X‐BT) and consumed a standardized diet during both study phases as described previously by our lab (Reynolds et al., 2015). Accelerometry data were not obtained in 1 older participant during the 3‐day NOEX phase, due to participant error. However, study staff confirmed with the participant that they did not exercise during that 3‐day removal of exercise phase. Further, as noted in Figures 1 and 3 participants were removed from the study due to inadequate CGM data. These subjects were missing meal postprandial glucose data for one or both phases of the study and thus analysis of our primary outcome of interest (1‐h PPIG) was not possible. Body composition was assessed via InBody Bioelectrical Impedance (InBody 770) to determine percent body fat and percent lean mass.

### 2.2 Participants

This study was approved by the Old Dominion University Institutional Review Board and written informed consent was obtained from all participants. Further, this research was done in accordance with the Declaration of Helsinki. This study was registered on Clinicaltrials.gov (ID: NCT04812392). Figure 1 represents a flow diagram outlining the study. Eleven older and nine young participants completed the study. Participants were recruited from the surrounding Hampton Roads, VA area. Subject characteristics are provided in Table 1. Inclusionary criteria for the participants were >55 years of age (old) or between 18–40 years of age (young), completing at least 30 min of moderate to vigorous exercise on 3 or more days per week during the past 3 months, and free from physical limitations that would interfere with daily physical activity. The American College of Sports Medicine defines current exercisers as those who participate in planned, structured physical activity of at least moderate intensity for 30 min per session on 3 or more days of the week during the past 3 months (American College of Sports Medicine, 2013); thus the participants recruited into this study were considered current exercisers. Exclusionary criteria included: weight change of </>3% within the previous 2 months, smoking within the previous 2 months, taking medications that alter blood glucose levels, HIV, hepatitis, tuberculosis, and hormone replacement therapy. The inclusion and exclusion criteria were determined via a self‐reported health history questionnaire.

**TABLE 1:** Demographics

Characteristic	Young	Old	*p*‐value
Age	33 ± 6	69 ± 7	<0.01
%body fat	24.7 ± 8.3	34.3 ± 5.4	<0.01
Skeletal muscle mass (kg)	32.4 ± 4.4	25.9 ± 5.3	<0.01
EX body weight (kg)	77 ± 8	73 ± 12	0.16
NOEX body weight (kg)	77 ± 8	72 ± 12	0.18
EX BMI (kg/m^2^)	25 ± 3	27 ± 4	0.41
NOEX BMI (kg/m^2^)	25 ± 4	26 ± 4	0.40
No of males/No of females	8 M/1 F	3 M/8 F	–
Energy intake (Kcals/day)	1988.9 ± 534.7	1741.6 ± 464.0	0.23
Carbohydrates (% Kcals)	45.3 ± 10.4	44.3 ± 10.0	0.83
Fat (% Kcals)	32.1 ± 10.7	32.3 ± 8.5	0.95
Protein (% Kcals)	17.4 ± 5.0	19.4 ± 4.9	0.52

*Note*: Data are represented as mean ± SD. Abbreviations: BMI, body mass index; EX, exercise phase; NOEX, removal of exercise phase.

### 2.3 Interventions

#### 2.3.1 Removal of exercise phase

During the removal of exercise phase (NOEX), participants refrained from completing exercise for 3 days. Further, participants were encouraged not to compensate for the removal of exercise by increasing their leisure time physical activity levels. An Actigraph accelerometer (wGT3X‐BT) was worn around the waist during the entire study intervention to determine steps and physical activity intensity.

#### 2.3.2 Accelerometry

**FIGURE 2:**
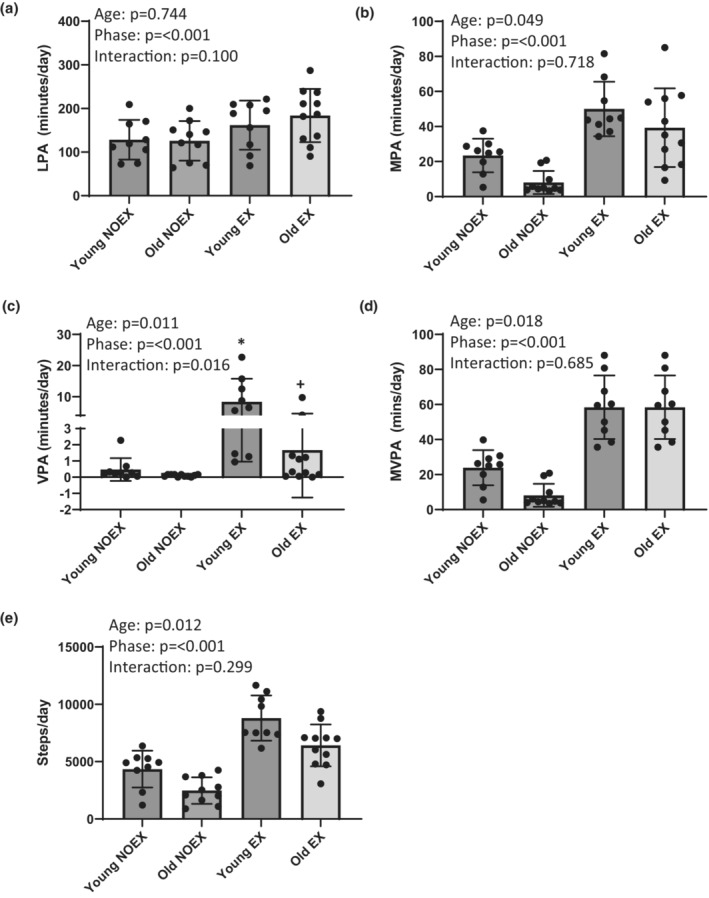
The 3‐day daily average of: (a) Light physical activity/day (LPA), (b) moderate physical activity/day (MPA), (c) vigorous physical activity/day (VPA) and (d) moderate‐to‐vigorous physical activity/day (MVPA) and (e) steps/day in young and older adults during 3‐days of habitual exercise (EX) and 3‐days of removal of exercise (NOEX). *p < 0.05 from the young no‐exercise phase (NOEX). +*p* < 0.05 from the young exercise phase (EX). Data are represented as mean ± SD.

**FIGURE 3:**
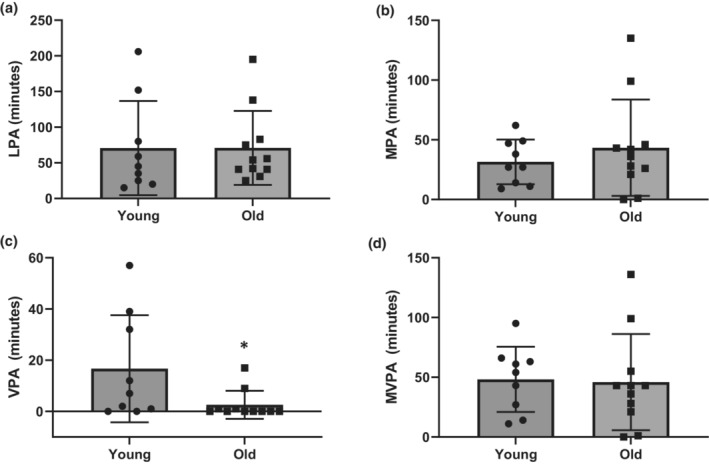
The 3‐day sum of: (a) Light physical activity/day (LPA), (b) moderate physical activity/day (MPA), (c) vigorous physical activity/day (VPA), and moderate‐to‐vigorous physical activity/day (MVPA) (d) in young and older adults. *p < 0.05 from the young group. Data are represented as sum ± SD.

Total daily steps acquired each day and total daily physical activity intensities (light physical activity (LPA), moderate physical activity (MPA), vigorous physical activity (VPA), and moderate to vigorous physical activity (MVPA)) were downloaded from the accelerometer and averaged across each 3‐day phase (Figure 2). Further, to determine exercise bout specific intensities during the 3‐day EX phase in the old and young subjects, accelerometry data were exported in 60‐s epoch length, and LPA (100–1951 counts), MPA (1952–5724 counts), and VPA (≥5725‐counts) were determined from Axis 1 counts for each minute of exercise and summed across the entire exercise bout (Freedson et al., 1998). Minutes of MVPA (≥1952 counts) was determined by summing the number of minutes of MPA and VPA during each exercise bout. Exercise bout specific intensities are reported in Figure 3.

#### 2.3.3 Body composition measurement

Body composition was assessed once during the study via bioelectrical impedance (InBody 770) following the removal of the glucose sensor after completing the EX or NOEX phase. Participants were instructed not to consume food or drink (other than water) for 4 h prior to testing or exercise for 24 h prior to the test. Further, participants provided a urine sample to assess hydration status, via urine‐specific gravity (USG). If USG was <1.025 then bioelectrical impedance was performed; this cut‐off has been used previously as a marker of euhydration (Thomas et al., 2016). No participants had USG > 1.025. Percent body fat and percent lean mass were recorded.

#### 2.3.4 Continuous glucose monitoring

**FIGURE 4:**
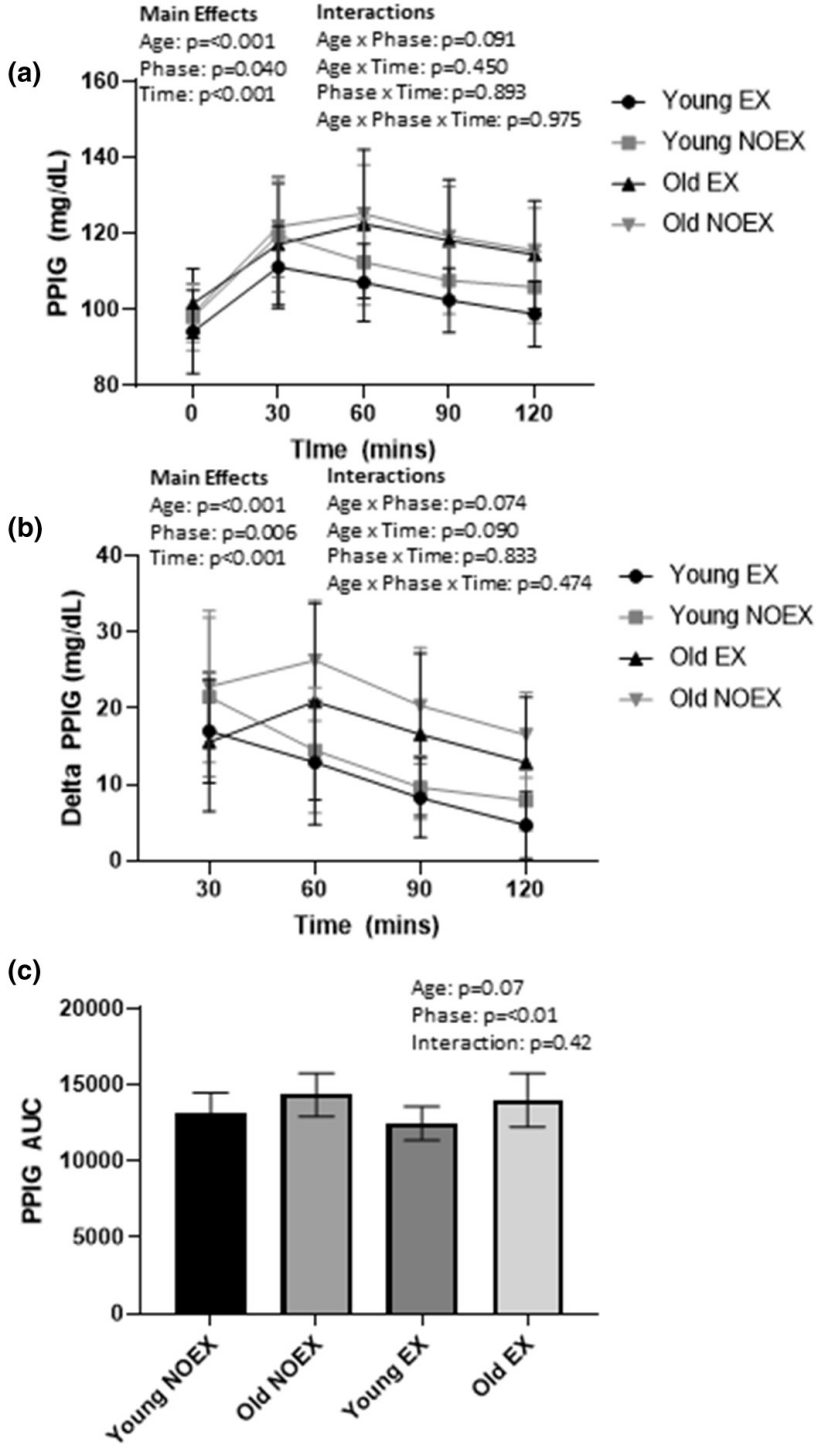
Postprandial interstial glucose (PPIG) (a), delta PPIG (b), and PPIG area under the curve (AUC) (c) in young and older adults 2 h following a meal during 3 days of habitual exercise (EX) and 3 days of removal of exercise (NOEX). Data are represented as mean ± SD.

Participants wore CGMS for 3 days during the removal of exercise and 3 days while performing habitual exercise. Briefly, the glucose sensor (Enlite™ glucose sensor, Medtronic Inc) was inserted subcutaneously into the abdomen approximately 3 inches to the right or left of the umbilicus on the day prior to the 3‐day monitoring period. A glucose monitor (iPro®2, Medtronic Plc.) was then connected to the sensor to store the glucose data. The iPro®2 CGMS collects interstitial glucose measurements every 5 min. Data were uploaded from the monitor to the Carelink website (carelink.minimed.com) to be processed. Raw data were then downloaded for analysis. Participants were asked to check their blood glucose levels using an Accu‐Chek glucose meter (Roche Diabetes Care, Indianapolis, IN, USA) four times each day during each study phase, record the values, and time of day via a log sheet. This was used to calibrate the CGMS. Participants were asked to perform their last bout of habitual exercise prior to 12:00 p.m. on the day before the NOEX phase. The primary measure was one‐hour Postprandial Glucose (PPIG). However, Emerson et al. (2018) also demonstrated significant 2‐h PPG responses between older active and young active adults. Thus, we also measured 2‐h PPIG. For completeness, peak PPIG, two‐hour PPIG area under the curve (AUC), 24‐h blood glucose standard deviation, and 24‐h maximum glucose values were also measured. All of these measures were determined from the CGMS. Glucose values were pooled across all 9 meals over 3 days, within each phase, for analysis (Figure 4 and Table 2). PPIG was calculated at 30‐min intervals up to 120 min after meal ingestion. Pre‐meal glucose values were reported as the glucose value which occurred 5 min prior to meal ingestion, as documented in the food log. Delta PPIG was calculated by subtracting pre‐meal glucose values from post‐meal values. Peak PPIG was calculated as the peak glucose value up to 120 min following a meal. The area under the curve was calculated at 15‐min intervals up to 120 min after meal ingestion. Similar to PPIG determination, pre‐meal glucose values were reported as the glucose value which occurred 5 min prior to meal ingestion.

**TABLE 2:** Three‐day average continuous glucose monitor data

	EX		NOEX		*p*‐value		
Measure	Young	Old	Young	Old	Group	Phase	Interaction
24‐h SD	10 ± 3	13 ± 5	13 ± 6	16 ± 4	0.09	0.01	0.72
Peak PPG	141 ± 16	148 ± 26	151 ± 29	159 ± 18	0.45	0.01	0.95
24‐h maximum	128 ± 8	143 ± 23	142 ± 21	149 ± 17	0.19	<0.01	0.18

Abbreviations: SD, standard deviation of glucose values; PPG, post prandial glucose.

#### 2.3.5 Dietary intake

To control for the effect of meal composition on blood glucose levels, participants consumed the same foods and volume of foods, and at the same time each day, over the course of each study phase. Further, participants were encouraged to wait at least 2 h between subsequent meals and/or snacks to allow for quantification of 2‐h PPIG. Participants were provided a food log to record the type, quantity, and time food was eaten each day during the first phase and then were asked to repeat that during the second phase. Study personnel verified that this was consistent between study days and phases by comparing the self‐reported food logs from each phase. Participants consumed food that was part of their normal, habitual, free‐living diet; food was not provided by study personnel. After collecting the food logs from participants, total energy, fat, protein, and carbohydrates consumed were determined using Cronometer (https://cronometer.com).

### 2.4 Statistical analysis

Statistical analyses were performed using SigmaPlot 12.5. Given that PPIG was our first variable of interest, we calculated our sample size utilizing 1‐h PPIG levels from Emerson et al. (2018). A sample size of 11 was needed for the trial to have 80% power to detect a two‐sided hypothesis test at an α level of 0.05 (effect size of 0.94) (G*Power, Version 3.1).

Differences in steps/day, LPA, MPA, VPA, MVPA, PPIG AUC, peak PPIG, glucose standard deviation, and maximum glucose values in the young and old adults during the EX and NOEX phases were detected using a 2‐way repeated measures anova (group × phase). Differences in 1‐h PPIG, 2‐h PPIG and delta PPIG across time, phase, and the group were detected using a three‐way anova. Non‐normally distributed data (PPIG and delta PPIG) were log‐transformed. Where statistical significance was found, least‐squared difference post hoc testing was applied. Statistical significance was set at *p* < 0.05. Data are expressed as mean ± SD.

## 3 RESULTS

Participant characteristics are listed in Table 1.

Steps/day and 3‐day, 24‐h averages of LPA, MPA, VPA, and MVPA minutes/day are represented in Figure 2. Both the old and young participants reduced their steps per day, LPA, MPA, VPA, and MVPA during NOEX compared to EX (*p* < 0.05). Further, the older adults took fewer steps/day and had fewer minutes of MPA, VPA, and MVPA/day compared to the young adults (*p* < 0.05). The only significant (*p* < 0.05) interaction found was within VPA. The young adults had reduced minutes of VPA in the NOEX phase compared to the EX phase and the old adults had less VPA in the EX phase compared to the young adults.

Old participants self‐reported that during the EX phase, they participated in walking/running (*n* = 9), cycling (*n* = 3), rowing (*n* = 3), swimming (*n* = 1), and resistance exercises (*n* = 5), and young participants self‐reported that they participated in walking/running (*n* = 7), cycling (*n* = 6), rowing (*n* = 1), elliptical (*n* = 1), and resistance exercises (*n* = 4). Exercise bout specific LPA, MPA, VPA, and MVPA summed across the 3 days of the EX phase in old and young participants are represented in Figure 3. The young participants participated in greater amounts of VPA compared to the old participants (*p* < 0.05). However, there was no statistically significant difference in LPA, MPA, or MVPA between the groups (*p* > 0.05).

Significant main effects of age (*p* = 0.002) and time (*p* < 0.001) existed for 1‐h PPIG, but no effect of phase or interactions (age × phase, age × time, phase × time, or age × phase × time) were found (*p* > 0.05). Glycemic control data are represented in Figure 4. Significant main effects of time, group, and phase (*p* < 0.05) exist for 2‐h PPIG and 2‐h delta PPIG; however, no significant interactions (age x phase, age x time, phase × time, or age × phase × time) (*p* > 0.05) were found. Thus, PPIG and delta PPIG were higher in the 2‐h postprandial period compared to pre‐meal. Further, the NOEX phase had higher 2‐h PPIG and 2‐h delta PPIG compared to the EX phase, and the older adults had greater 2‐h PPIG and 2‐h delta PPIG compared to the young adults. 2‐h PPIG AUC had a trend (*p* = 0.07) for significant effects of age and significant (*p* < 0.01) effects of phase but no phase‐by‐age interaction was found (*p* > 0.05). Table 2. demonstrates other glycemic control variables. Peak PPIG, glucose standard deviation, and maximum glucose values were significantly higher in the NOEX phase compared to the EX phase (*p* < 0.05); however, no main effects of group or interaction were found with any of these variables.

## 4 DISCUSSION

Three days of exercise removal impairs glycemic control in older adults as well as young adults. Twenty‐four‐hour blood glucose standard deviation, an indicator of the variability of blood glucose concentrations, and 24‐h maximum glucose values were both elevated in the NOEX phase compared to the EX phase, suggesting greater fluctuations in blood glucose. Further, peak PPIG, mean PPIG, PPIG AUC, and delta PPIG were also higher during the NOEX phase compared to the EX phase. PPIG and delta PPIG were also higher in the older adults compared to the young adults, yet no interaction was found. Collectively, these results suggest that an acute decrease in habitual exercise and older age worsen glycemic control; and the detriments in glycemic control are consistent between young and old adults in response to the removal of exercise. This is clinically relevant and adds to the body of evidence supporting the use of physical activity and exercise in preventing cardiovascular disease in aging and older adults (Jakovljevic, 2018).

Exercise is widely known to have numerous health effects, particularly in regard to metabolic health (Thyfault & Bergouignan, 2020; Wake, 2022). As demonstrated in the classic study by Mikines et al (Mikines et al., 1988), as little as a single bout of exercise in sedentary (young) individuals improves insulin sensitivity. Further, specifically in older adults, short‐term exercise training improves insulin sensitivity (Bloem & Chang, 2008). The American College of Sports Medicine recommends that all adults participate in at least 150 min of moderate‐intensity or 75 min of vigorous‐intensity exercise each week (Haskell et al., 2007). Unfortunately, approximately half of the individuals living in the United States do not meet these minimum criteria (Centers for Disease Control, 2018), and adults >50 years old make up ~30% of those individuals who are physically inactive (Watson et al., 2016). We and others demonstrate that transitioning from taking >10,000 steps/day to <5000 steps/day for 3–5 days impairs glycemic control in young, active adults (Mikus, Oberlin, Libla, Taylor, et al., 2012; Reynolds et al., 2015). In this study, both the old and young subjects reduced total daily steps in the NOEX phase by >50% from the EX phase. Subsequently, PPIG was also elevated during the NOEX phase compared to the EX phase. While reducing physical activity is different from the removal of exercise, these studies support the findings of the present study that the removal of exercise increases PPIG in active older adults.

Numerous studies demonstrate the importance of physical activity and exercise on PPG in young adults (Coe et al., 2018; Kurti et al., 2022; Mikus, Oberlin, Libla, Taylor, et al., 2012; Reynolds et al., 2015). However, limited research exists on older adults. Emerson et al. (2018) examined blood glucose responses following a standardized meal in the laboratory in older active adults compared to older inactive adults and found that the older active adults had elevated 1‐h blood glucose levels compared to the young active adults. Further, the older inactive adults had elevated 1‐h blood glucose levels compared to older active adults. This study did not have a young inactive adult group. The results of this study support the findings of the present study, and we extend these findings by (1) determining the acute alterations in free‐living glycemic control response to short‐term removal of exercise in older adults, and (2) comparing that response to young adults. In the present study, older adults accumulated fewer minutes of vigorous‐intensity activity during exercise compared to young adults. However, both groups completed the same amount of light, moderate, and moderate to vigorous physical activity. Thus, it is possible that if the older adults participated in the same levels of vigorous‐intensity exercise as the young adults, they might have experienced an even greater improvement in glycemic control variables during the EX phase. However, this is speculative and requires further research. Coker et al. (2006) demonstrated that older adults do not improve insulin‐stimulated glucose disposal following moderate‐intensity exercise training; rather, only vigorous exercise training improved insulin‐stimulated glucose disposal. However, Cox et al. (1999) found similar improvements in whole‐body insulin sensitivity in young and old participants following 7 days of moderate‐intensity exercise training. While the physiological adaptations when transitioning from a sedentary state to an exercise‐trained state are not expected to merely be the opposite as when transitioning from the exercise‐trained state to the sedentary state, it is clear that more research is needed to determine the impact of exercise intensity on negating impairments of glycemic control on the removal of exercise.

Oscillations in blood glucose levels have been demonstrated to have deleterious outcomes on flow‐mediated dilation, a measure of endothelial function that has been demonstrated to predict cardiovascular events (Matsuzawa et al., 2015). Compared to sustained hyperglycemia, severe oscillations, or glycemic variability, seem to be more harmful (Ceriello et al., 2008). Ceriello et al. (2008) demonstrated that oscillating blood glucose levels resulted in poorer endothelial function and greater oxidative stress levels in the blood compared to sustained hyperglycemia. These data support the notion that glycemic variability is an important predictor of cardiovascular disease. The 24‐h standard deviation of blood glucose levels around the mean is a common measure to assess glycemic variability. This measure assesses the variability in the major and minor fluctuations in blood glucose levels (Monnier et al., 2008). In the present study, blood glucose standard deviation was elevated during NOEX compared to EX, but no significance was found when comparing this in old vs. young adults. Nonetheless, these results demonstrate the importance of daily exercise on glycemic variability in both older and young adults.

Prolonged, excessive caloric intake may negatively impact insulin sensitivity (Samocha‐Bonet et al., 2012). However, studies demonstrate that reducing energy intake during 1 day of prolonged sitting does not fully prevent impairments in insulin sensitivity in response to acute inactivity (Stephens et al., 2011). Thus, impairments in insulin sensitivity that occur in response to short‐term inactivity may not be solely due to excessive caloric intake. On the contrary, some studies demonstrate that short‐term overfeeding does not impair PPG or insulin sensitivity. Adochio et al. (2009) found that overfeeding (+40% energy) for 5 days does not appear to impair insulin sensitivity. Likewise, Morrison et al. (2019) demonstrated that 5 days of overfeeding (+45% energy) did not change postprandial glucose and insulin responses. However, in the present study, participants consumed the same amount of energy in the EX‐phase compared to the NOEX phase. Thus, while the impact of short‐term overfeeding during inactivity on PPG is not fully understood, we cannot fully exclude the potential role of excess caloric intake during the removal of the exercise phase on glycemic control. However, this model does appear to simulate real‐world scenarios of the interactions between sedentary behavior and caloric intake (Bassett Jr. et al., 2010).

Importantly, it is also necessary to address that the CGM measures interstitial glucose levels not blood glucose levels. Much of the literature examining detrimental health links to PPG examined blood glucose (Cavalot et al., 2006; Fuller et al., 1980; Pyorala, 1979). While under steady‐state conditions interstitial and blood glucose levels have been found to be similar (Lonnroth et al., 1987), studies demonstrate mixed results on whether blood glucose and interstitial glucose levels are similar during exercise (Figueira et al., 2012; Heden et al., 2018; Yardley et al., 2013). Herrington et al. (2012) demonstrated, in women, that interstitial glucose levels during steady state cycling exercise were elevated compared to blood glucose levels. While Yardley et al. (2013) did not observe differences in interstitial glucose levels compared to blood glucose levels in individuals with type 2 diabetes during steady state running exercise. This is an area of research that requires much greater investigation to tease apart the driving factors behind these differences. Nonetheless, it is possible that during the EX phase of the present study, the glucose values reported are higher than the “true” blood glucose values which may minimize the differences between EX and NOEX phases.

Some limitations of this study exist. The sample size of the study was small limiting the generalizability of the findings. Further, the groups were not evenly matched by gender. The young participants had a greater number of men than women and the older participants had a greater number of women than men. It is important to point out that the older women were all post‐menopausal, which may minimize the hormonal differences (mainly estrogen) between the groups. Pre‐menopausal women tend to have greater insulin sensitivity and greater insulin‐mediated glucose disposal, compared to men, likely due to the insulin‐sensitizing effects of estrogen (De Paoli et al., 2021; O'Sullivan & Ho, 1995). However, insulin sensitivity and insulin‐mediated glucose disposal do not appear to be different in post‐menopausal women compared to young or old men (Nilsson et al., 2000). Further, the data in the present study confirm what others have shown, in that older adults have decreased skeletal muscle mass and increased fat mass compared to young adults. While age‐related loss of muscle mass and increased adipose tissue exists (Koster et al., 2011; Lexell et al., 1988); we cannot fully exclude the possibility that this is what is driving the age‐related differences in glycemic control in the present study and whether these differences would remain if the groups were matched by skeletal muscle mass and adiposity. Yet, matching based on skeletal muscle mass is not practical and may detract from clinical relevance due to the presence of the described body composition differences between older and young adults. Nonetheless, both aging and increased obesity are related to inflammatory conditions which impair insulin sensitivity and are associated with reduced skeletal muscle mass (Park et al., 2007; Schaap et al., 2009; Schrager et al., 2007). Thus, while there are anticipated body composition differences in the young compared to the old subjects, results from this study may more accurately describe real‐world implications of aging on free‐living glycemic control. Further, exercise time in relation to meal consumption was not controlled for in this study. Studies demonstrate that the timing of exercise impacts postprandial glucose responses (Aqeel et al., 2020). Future studies should aim to control this. Lastly, habitual exercise prior to the study intervention was determined via self‐report. Self‐reported physical activity has been shown to overestimate measured physical activity levels (Prince et al., 2008), which is represented in our data set where older and younger adults self‐reported longer duration bouts per habitual exercise session compared to what was measured from the accelerometer during the 3‐day habitual exercise study intervention.

To conclude, we demonstrate that while older adults experience worse glycemic control than younger adults, and that short‐term removal of exercise impairs glycemic control, older adults do not experience a greater impairment in glycemic control compared to young adults. Our data support the overall need for regular, daily exercise in adults, particularly older adults, who have a worse glycemic profile.

## CONFLICT OF INTEREST

The authors report no conflicts of interest. The results of the study are presented clearly, honestly, and without fabrication, falsification, or inappropriate data manipulation.
